# Supplementary material for: Sound Absorption Improvement in Porous Ferroelectret Polyethylene with Effective Piezoelectric Mechanism
Source: Polymers (Basel). 2022 Nov 10;14(22):4843. doi: 10.3390/polym14224843 (PMC9697414; doi:10.3390/polym14224843)
Supplement: Supplementary file 1 [file polymers-14-04843-s001.zip › polymers-1985843-supplementary.pdf]

Supplementary Material

Sound Absorption Improvement in Porous Ferroelectret Polyethylene with Effective Piezoelectric Mechanism

Yasmin Mohamed Yousry [1,†], Eleftherios Christos Statharas [1,2,†], Kui Yao [1,*], Ayman Mahmoud Mohamed [1], Poh Chong Lim [1] and Francis Eng Hock Tay [2]

1 Institute of Materials Research and Engineering, A*STAR (Agency for Science, Technology and Research), 2 Fusionopolis Way, Innovis, Singapore 138634, Singapore
2 Department of Mechanical Engineering, National University of Singapore, 9 Engineering Drive 1, Singapore 117575, Singapore
* Correspondence: k-yao@imre.a-star.edu.sg; Tel.: +65–64168936
† These authors contributed equally to this work.

Sound absorption coefficient of the PE foam samples was measured according to the ASTM E1050-08 procedure through an acoustic tube (Bruel & Kjaer 7758). The foam samples were activated as ferroelectret with effective piezoelectric property using electrical charging by a corona poling setup, as shown in Figure S1. The effect of corona poling on the morphology and microstructure of the PE foams was examined with field emission scanning electron microscopy (FESEM: JSM-6700F, JEOL), as shown in Figure S2. The effect of corona poling and annealing on the mechanical properties of the foam samples were examined with a dynamic mechanical analyzer (DMA Q800), as shown in Figure S3 and S4. The dielectric properties of the samples were measured using an impedance analyzer (Agilent 4294 A), as shown in Figure S5.

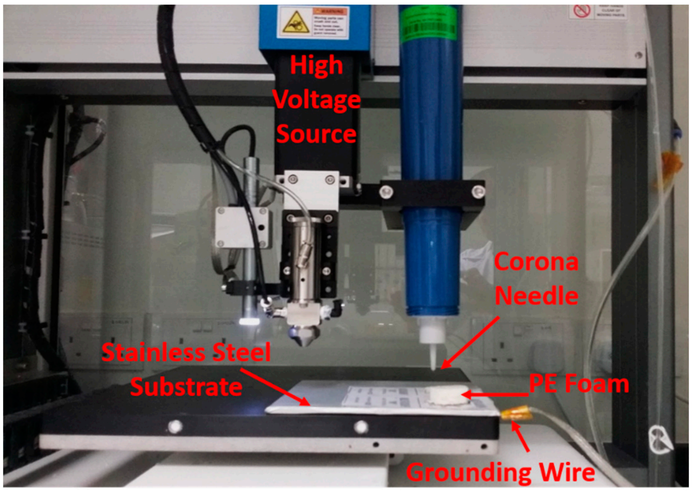

Figure S1. Photo of the corona poling setup.

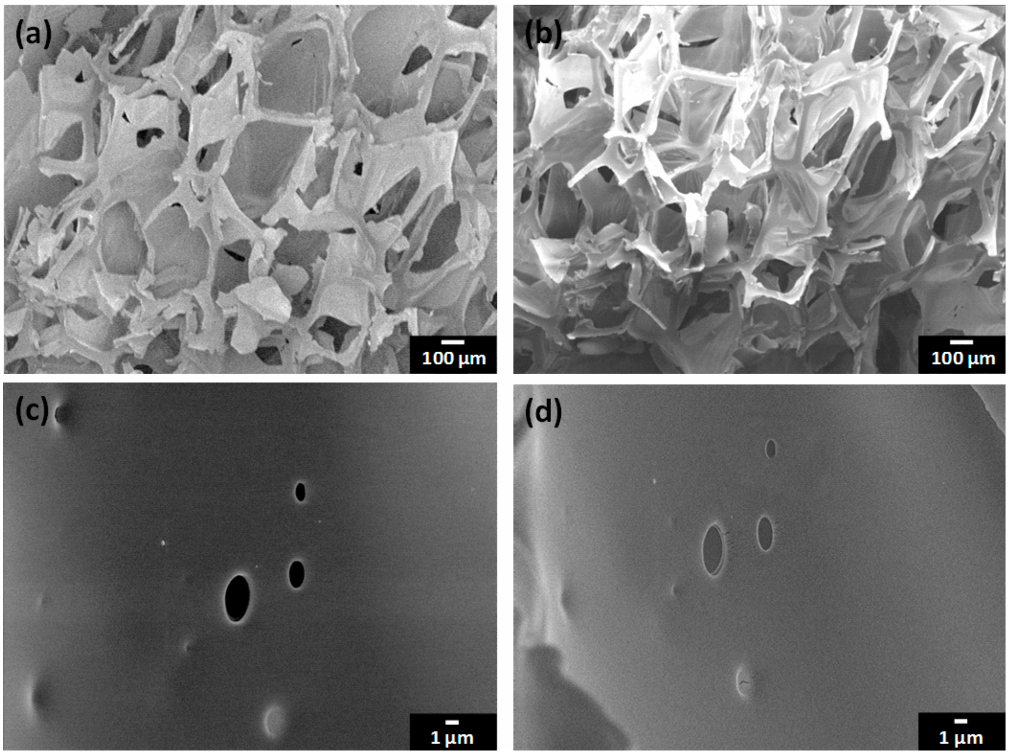

Figure S2. FESEM micrographs of PE foam samples before (a,c) and after (b,d) corona poling at 55 kV for 3 minutes.

Figure S3 (a) and (b), supplementary material, present the measured stiffness of PE foam samples with and without corona poling before and after annealing treatment at 80 °C for 1 hour, respectively. The results show very minor effects on the stiffness of the foams from the corona poling or annealing.

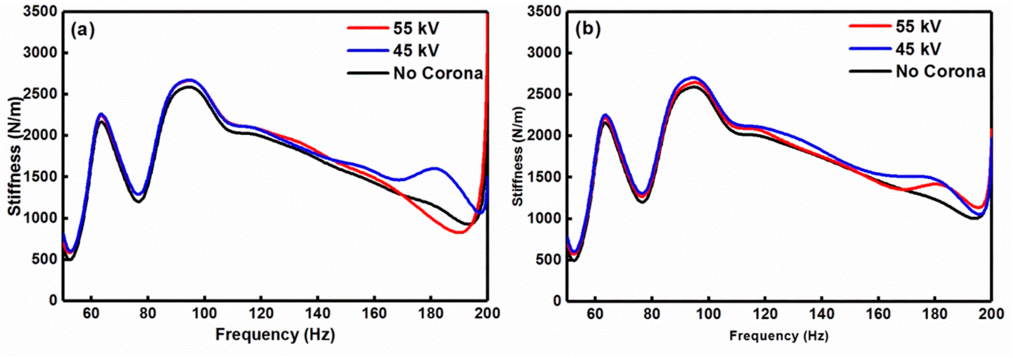

Figure S3. Stiffness of PE foam samples with and without corona poling before (a) and after (b) annealing at 80°C for 1 hour.

Moreover, the tan delta of the foams with and without corona poling and before and after annealing can be seen in Figure S4(a) and (b). The tan delta is a property that shows the mechanical energy loss of the foam and is calculated from the ratio of loss modulus to the storage modulus. The results show very minor effects on the tan delta of the foams from the corona poling or annealing at 80 °C.

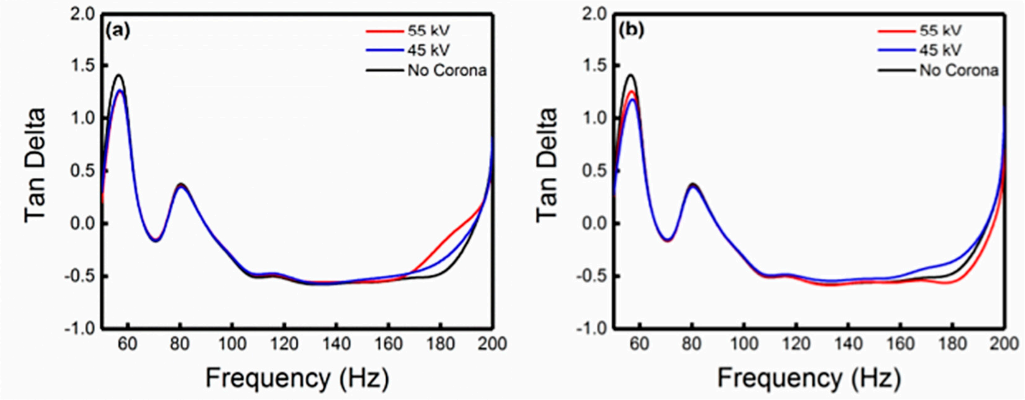

Figure S4. Tan Delta of PE foam samples with and without corona poling before (a) and after (b) annealing at 80°C for 1 hour.

In contrast, corona poling and annealing treatments significantly changed the dielectric and piezoelectric properties of the PE foams. As shown in Figure S5(a), supplementary material, where the dielectric constant of 2.5 of the original PE foam sample increased to 3.2 after poling with 45 kV and 3.7 with 55 kV. The dielectric constant returned to almost the same value of 2.5 after annealing at 80 °C as shown in Figure S5(b).

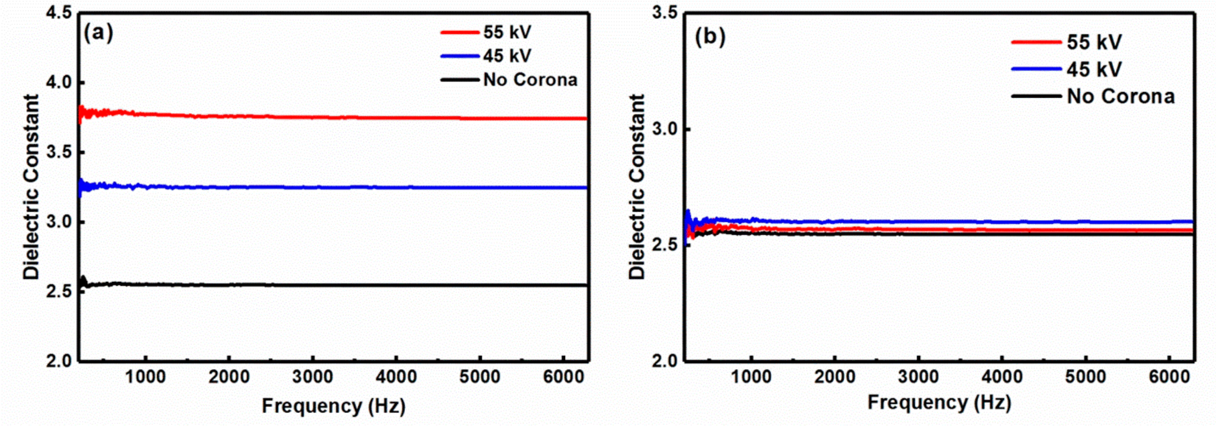

Figure S5. Dielectric constant of PE foam samples with and without corona poling before (a) and after (b) annealing at 80°C for 1 hour.
